# Total Knee Arthroplasty in Severe Valgus Osteoarthritis Excellent Early Results in a 90-Year-Old Patient with a Valgus Deformity of 47°

**DOI:** 10.1155/2017/9301017

**Published:** 2017-03-12

**Authors:** Petros Ismailidis, Rolf Kernen, Sebastian Andreas Mueller

**Affiliations:** ^1^Department of Orthopedic Surgery, University Hospital of Basel, Spitalstrasse 21, 4056 Basel, Switzerland; ^2^Clinic for Orthopedics “Claraortho”, Claragraben 82, 4058 Basel, Switzerland

## Abstract

Grade III valgus deformity (tibiofemoral alignment > 20°) is present in only 0.5% of patients receiving total knee arthroplasty. Furthermore, cases with a valgus deformity exceeding 40° are even rarer. Since they mostly affect elderly, polymorbid patients, successful outcome means a great challenge. We report on a case of a 90-year-old patient with a valgus deformity of 47°. The patient was preoperatively restricted to a wheel chair, unable to walk, and only able to stand for a few seconds. The maximal knee flexion was 100°, and there was an extension deficit of 15°. The WOMAC score was 91; the EQ-5D-5L Index was 0.048. She was treated with a constrained hinged prosthesis. Postoperatively, the axis was 6° valgus. After 3 months of rehabilitation, she was independent using a wheeled walker. The maximal flexion of the knee was 110° and there was no extension deficit. The WOMAC score was 45; the EQ-5D-5L Index was 0.813. This case demonstrates the possibility of a satisfactory result and an improvement in quality of life and mobility with a plausible timetable and with reasonable use of resources even in advanced age and severe valgus deformity.

## 1. Introduction

Valgus knee on standing anteroposterior knee radiographs is present in nearly 10% of the patients undergoing total knee arthroplasty (TKA) [1]. Valgus deformity is classified in 3 grades: grade I (80% of cases) with an axial deviation of 6–10°, passively correctable, with contracture of the lateral soft tissue but without elongation of the medial collateral ligament (MCL); grade II (15% of cases) with an axial deviation of between 10° and 20°, where the lateral structures are contracted and the MCL is elongated but sufficient; grade III (5% of cases) with an axial deformity greater than 20°, tight lateral structures, and insufficient medial stabilizers [2]. The more the deviation and thus the instability have to be corrected, the more constraint the implants need to be. However, the required constraint is subject of debate, although there is a general agreement that the least amount of constraint providing a stable knee should be used. Mostly in cases of grade I or II deformities [1, 3] but even in grade III deformities [4], good results have been published with posterior cruciate retaining (CR) [3] or posterior stabilized (PS) [1, 3, 4] prostheses. However, in most cases with grade III deformity where the medial knee structures are completely nonfunctional, a greater amount of constraint should be used [1, 2]. A satisfying result in grade III valgus deformities is challenging even for experienced surgeons.

Successful TKA in geriatric patients is challenging. Geriatric patients have been shown to have a higher rate of intra- and postoperative complications and longer hospitalization [5, 6]. An age of 70 years or older is a predisposing factor for postoperative delirium [7], the incidence of which is up to 50% after orthopedic operations [8]. Furthermore, the rehabilitation time can be prolonged because of the comorbidities and preexisting strength deficit. Up to 38% of quadriceps strength deficit has been reported in end stage osteoarthritis of the knee [9], while in geriatric patients with severe osteoarthritis this deficit is expected to be even larger. Altogether, patients above 80 years have been shown to have poorer general outcomes following arthroplasty [6]. Currently only 5% of all TKAs are performed in patients aged 85 years or older [5].

Both extreme valgus deformities and advanced patient age are risk factors for complications and poor results after TKA. Severe deformities often evolve over time, and geriatric patients already lack good mobility with relevant muscle degradation before operation. Clearly, this has a negative influence on the rehabilitation time and result. For the above reasons, TKA in patients older than 80 years and with a valgus deformity >40° is very seldom performed.

## 2. Case Presentation 

**Figure 1:**
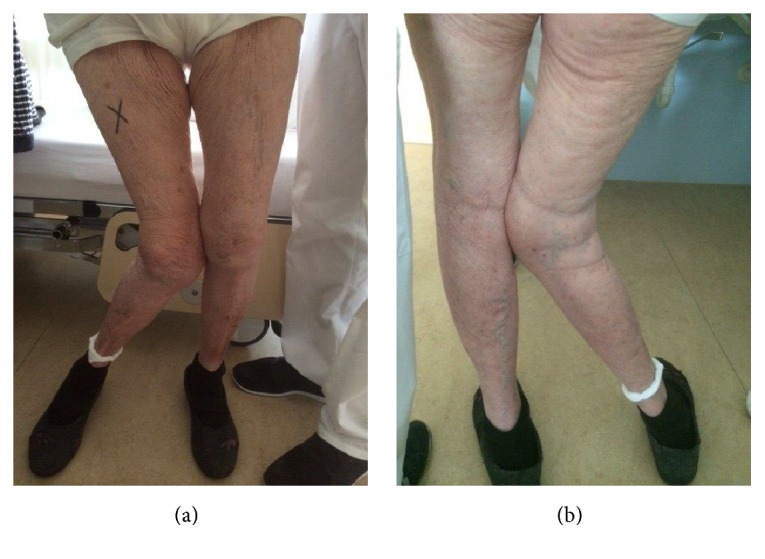
Preoperative anterior (a) and posterior (b) view images of the patient standing. A massive valgus knee deformity can be inspected.

**Figure 2:**
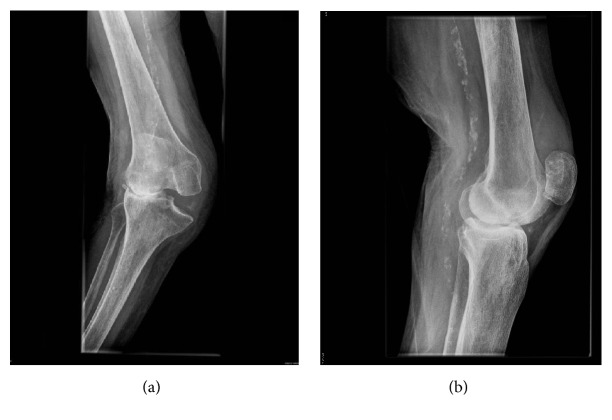
Preoperative standing X-rays of the knee: anteroposterior (a) and lateral (b) view. There is a valgus alignment of 47° (grade III valgus osteoarthritis). Significant wear and destruction of the lateral condyle.

We report on the case of a TKA of a 90-year-old female patient with a grade III valgus arthritis with a valgus angle of 47° (Figures 1 and 2). A written patient consent was received and archived preoperatively. An ethical approval was not required. The patient was presented in our outpatients' clinic unable to walk even with an aid and able to stand with support only for a few seconds with severe pain in the knee. The valgus deformity had evolved gradually over the course of the previous 6 months and the patient was restricted to a wheel chair over that period. As a consequence, the patient has had severe atrophy of her quadriceps muscles. There was complete instability in the mediolateral plane with fully nonfunctional medial knee stabilizers and tight lateral structures. There was no peripheral vascular or neurological deficit. The WOMAC score (Western Ontario and McMaster Universities Arthritis Index) [10] on presentation was 91; the EQ-5D-5L Index (European Quality of Life Five-Dimension Five-Level Scale) [11] was 0.048. The maximal flexion was 100°, and there was an extension deficit of 15°. The patient was living in a retirement home. The comorbidities included a mild dementia with a score of 24/30 in the Mini-Mental State Examination (MMSE) [12]. Regarding the surgery risk, the patient was classified as ASA 2 [13].

The WOMAC is a widely used score to determine outcome and consists of 24 questions covering 3 dimensions: pain (5 questions), stiffness (2 questions), and function (17 questions). The score ranges from 0 to 100 (higher scores indicating poor results) [10]. The EQ-5D-5L Index is a standardized instrument to subjectively measure a health condition. It consists of questions covering 5 dimensions of health condition (mobility, self-care, usual activities, pain/discomfort, and anxiety/depression) each in five levels. According to the answers, an index of health status can be calculated ranging from 0 to 1, with 1 being the best possible health condition [11].

The operation was conducted under touniquet under spinal anesthesia and a peripheral femoral nerve block. A cemented constrained hinged knee prosthesis (Zimmer® NexGen® RH Knee) was implanted, through an anterolateral parapatellar skin incision with a medial parapatellar arthrotomy. Tibial resection was conducted at 90° to the tibial axis using an intramedullary instrumentation. Distal femoral resection was conducted in 6° of valgus alignment. Only a minimal amount of lateral femoral condyle was removed because of preexisting wear and destruction. Lateral collateral ligament and popliteus tendon were released, in order to correct the fixed valgus deformity and restore the joint line. A 12 mm inlay was used. The patella was not replaced. Intraoperatively complete joint stability could be achieved with an extension of 0° and a flexion of 110°.

Postoperative pain management included a multimodal drug therapy with continuous infusion and repeated boluses of local anesthetic through the femoral catheter, as well as oral analgetics. The mobilization of the patient began on the first postoperative day with full weight bearing. The inpatient acute care lasted for two weeks, followed by two weeks of inpatient extended care in a rehabilitation clinic and another two months of physical therapy twice a week after being discharged to the retirement home. Physical therapy was focused during the first days on achieving full extension and flexion up to 90° with the use of a CPM (continuous passive motion) machine as well as consequent edema control, followed by upper body training, gait training, and balance/proprioception exercises. Logically, after six months of solely wheel chair mobilization and taking into consideration the comorbidities (mild dementia), the rehabilitation time was prolonged. There was mild postoperative delirium with a Delirium Observation Screening (DOS) scale [14] of 6/13 on the first and 3/13 on the second postoperative day and in the normal range (<3) thereafter. The treatment required the coordinated effort of the orthopedic surgeon, anesthetist, physical therapist, case manager, and nursing staff.

**Figure 3:**
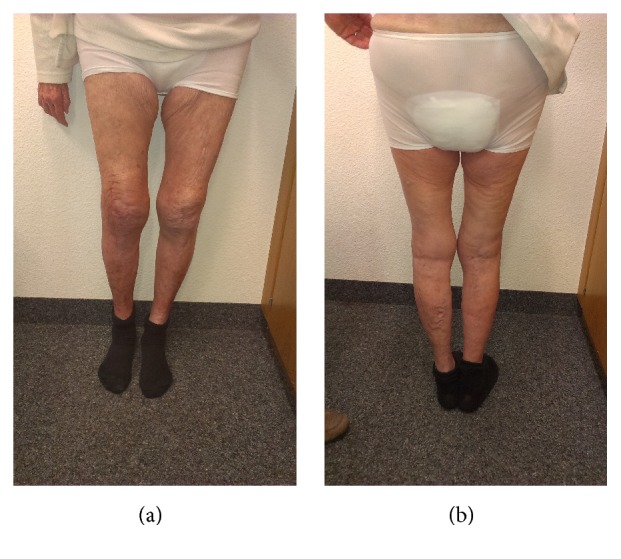
Postoperative anterior (a) and posterior (b) view images of the patient standing. Visual inspection with normal tibiofemoral alignment.

**Figure 4:**
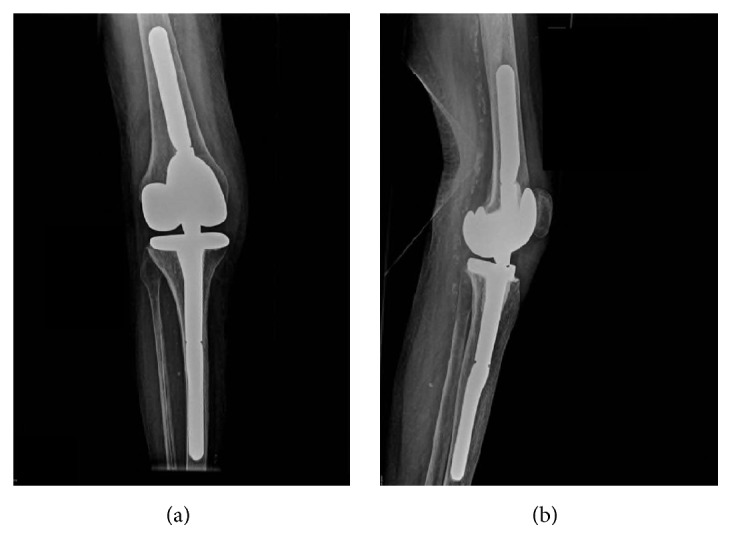
Postoperative standing X-rays of the knee: anteroposterior (a) and lateral (b) view. Tibiofemoral alignment of 6° valgus.

**Figure 5:**
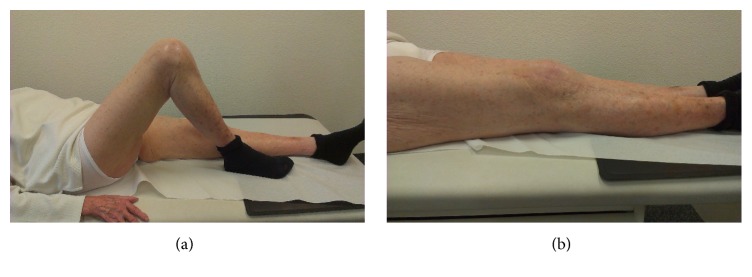
Postoperative Range of Motion (ROM) flexion (a) of 110° and extension (b) 0°.

The complete rehabilitation regime for this patient lasted for three months. After that time, the patient presented in our outpatients' clinic able to walk for about 200 meters using a wheeled walker. She had regained the level of activity she previously had in the retirement home one year preoperatively. She had no knee pain and received no pain medication. The WOMAC score improved to 45 and the EQ-5D-5L Index improved to 0.813. The tibiofemoral alignment in the standing X-rays was 6° valgus (Figures 3 and 4). There was no sagittal or mediolateral instability in the clinical examination. The maximal flexion was 110° and there was no extension deficit (Figure 5).

## 3. Discussion

There are no age restrictions or deformity restrictions for TKA. However, both extreme age and extreme deformity are unfavorable factors for the end result. The recent changes in patient demographics with an increasing life expectancy are supposed to raise the number of TKA in both geriatric patients and valgus knees.

Due to the technical demand of the operation in severe valgus deformity and the increased risk of complications in the elderly patients, most surgeons indicate the operation rather reluctantly. When additionally considering the prolonged rehabilitation time at an old age, the real benefit to the patients in a reasonable time frame can be questioned. Even in studies reporting correction of valgus deformities involving large number of patients (Table 1), correction of deformities above 40° is rarely described. In the few cases reported, patients with a valgus deformity greater than 40° did not belong to the geriatric population. To the best of our knowledge, Zhou et al. [15] reported the highest mean preoperative tibiofemoral valgus angle (33°  ± 9.7°; 21–52°) in the literature. However, this population was much younger with a mean age of 57 (47 to 63 years). We did not find any case presenting a patient older than 80 years with a valgus osteoarthritis of the knee >40 treated with TKA.

In conclusion, this case demonstrates that even extreme tibiofemoral alignment deformities in advanced age should not be considered as absolute contraindication for TKA. However, the indication and the patient should be evaluated thoroughly with respect to comorbidities and expected risk profile. We believe that the intense cooperation of patient, relatives, treating physicians, social services, nursing staff, and physical therapists is mandatory and thus can lead to a considerable improvement of quality of life within a legitimate timeframe and with reasonable use of resources.

## Figures and Tables

**Table 1:** Studies reporting TKA in valgus osteoarthritis showing the mean valgus and patient age.

Study	Number of knees	Mean valgus alignment	Range of valgus	Mean age of patients in years	Range of patient age in years
Lombardi Jr. et al [3].	97	15°	10−31°	66	42−88
Ranawat et al. [1]	42	15°	Not reported	Not reported	Not reported
Zhou et al. [15]	32	33°	21−52°	57	47−63
Apostolopoulos et al. [4]	24	23°	15−35°	72	57−79
Karachalios et al. [16]	34	27°	20−40°	Not reported	Not reported
Sekiya et al. [17]	47	14°	6−24°	65	50−77
Chalidis et al. [18]	57	11°	10−17°	71	45−77
Hadjicostas et al. [19]	15	21°	17−27°	73	64−80
Bremer et al. [20]	79	20°	8−40°	71	Not reported
Boyer et al. [21]	63	15°	10−27°	65	26−81
Mullaji and Shetty [22]	10	19°	10−37	65	48−77
